# Economic Synergy between Dry Cow Diet Improvement and Monensin Bolus Use to Prevent Subclinical Ketosis: An Experimental Demonstration Based on Available Literature

**DOI:** 10.3389/fvets.2017.00035

**Published:** 2017-03-14

**Authors:** Didier Raboisson, Maxime Barbier

**Affiliations:** ^1^IHAP, INRA, ENVT, Université de Toulouse, Toulouse, France

**Keywords:** subclinical ketosis, economics, cost, monensin bolus, profitability

## Abstract

The prevention of subclinical ketosis (SCK) is based on maintaining adequate nutrition in dairy cows during the dry period and close to calving. Recently, an oral-route monensin bolus to prevent SCK was approved in Europe. The present study aims to define the allocation of resources for SCK management at the herd level and evaluate the profitability of administering monensin boluses in cows at risk for SCK. A stochastic model was used to calculate the total cost of SCK for a population with a given prevalence of cows at risk for SCK. This model included the ability of the farmer to correctly target and preventatively treat these cows at risk for SCK. The results clearly demonstrated economic synergy between two management practices. First, reducing the prevalence of cows at risk for SCK dramatically reduces the total cost of SCK and seems profitable in most situations. Second, monensin bolus use to reduce the occurrence of SCK in cows already at risk for SCK is cost-effective. The results also highlighted three economic strategies to manage SCK in the dairy industry in Europe. First, monensin bolus use throughout an entire herd when the prevalence of cows at risk for SCK is high is only profitable in the short-term as a tool to correct acute deterioration at the herd level. Second, decreasing the prevalence of cows at risk for SCK through adequate feeding in the dry period is of financial interest as a baseline strategy when prevalence is high, assuming moderate additional cost linked to the new diet. Third, monensin bolus use when the prevalence of cows at risk for SCK is low is also profitable as a long-term strategy when only cows at high risk for SCK (such as cows that are over-conditioned, old, or have a previous history of SCK-related disorders) are targeted for preventative treatment. Authors suggest to use the present results considering that farmers have a correct, but not perfect, ability to target animals to be preventively targeted with the monensin bolus. Further work is required to facilitate the early identification of cows at risk for SCK.

## Introduction

Subclinical ketosis (SCK) is a common metabolic disorder in dairy cows caused by a strong dietary negative energy balance around or after calving. A certain degree of negative energy balance is expected in cows during late gestation and early lactation; however, SCK occurs if the blood ketone concentration increases dramatically (1). The beta-hydroxybutyrate (BHBA) threshold for SCK diagnosis is generally between 1.2 and 1.4 mmol/l (2). Increased concentrations of both BHBA and non-esterified fatty acids are markers of SCK. As a result of the imbalance between energy expenditure and ingestion, SCK is not only observed in high-producing dairy cows but also in cows with moderate milk production and inadequate ingestion or feeding around calving. The lactational prevalence of SCK in Europe is estimated to be between 25 and 47% (3, 4) and up to 85% of herds are reported to have an intra-herd prevalence of more than 25% (5).

Subclinical ketosis has both societal and economic implications. First, SCK is associated with an increased risk for the majority of health disorders (puerperal metritis, purulent vaginal discharge, placental retention, abomasum displacement, mastitis, and lameness) observed in dairy cows during the early postpartum period (2). Consequently, SCK leads to increased antimicrobial use. The application of antimicrobials in herds with a low prevalence of SCK compared with herds with a moderate prevalence of SCK is, on average, 10 or 25% less frequent, depending on circumstances (6). SCK has a large impact on farm profitability. The total average cost of SCK in Europe was recently estimated to be €257 (i.e., US $283) per cow with SCK (95% prediction interval = €74–442; US $81–487) (7). Decreased milk production during SCK only accounts for 11% of the total cost. Early culling and abomasum displacement are the two major contributors of SCK total cost. Furthermore, SCK total cost sensitivity to the feed cost margin is low.

Subclinical ketosis prevention is based on maintaining adequate nutrition in dairy cows during the dry period. The most important risk factor during this period is over-conditioned cows, which leads to fat cows at calving and reduced dietary intake around calving and during the early postpartum period (1, 8). Well-known methods to prevent SCK include adequate feeding of dry cows including adequate energy density diets, adequate management of early lactation diets, with for instance improved ingestion through adequate comfort of animals. Despite this, many cows remain at risk for SCK during the few weeks prior to calving. It is sometimes difficult to offer adequate diets to certain cows, such as those grazing in pastures. In particular, small farms face difficulties in preparing an appropriate diet for low numbers of dry cows, often just one or two cows during the transition period. Regardless of the cause, the prevention of SCK is primarily based on a cow’s *prepartum* diet. Until recently in Europe, there was no preventative solution for cows that were fat at several weeks prepartum, and weight reduction at this stage increases the risk of developing SCK. Recently, an oral-route monensin bolus was authorized in Europe to prevent SCK when administered 3 weeks prior to calving in cows at risk for SCK. This drug has been intensively used outside of Europe (9, 10). In Europe, monensin is labeled for use in prepartum cows that are at risk for SCK, which are defined as cows that are over-conditioned, old, and have a history of SCK-related disorders during previous lactations^1^.

Therefore, the aim of the present study was to define (i) resources to be allocated for SCK management at the herd level without monensin bolus use and (ii) the profitability of monensin bolus use in cows at risk for SCK. Using economic modeling, this study sought to define optimal situations in terms of SCK-related herd management.

## Materials and Methods

### Concept and Model

**Figure 1:**
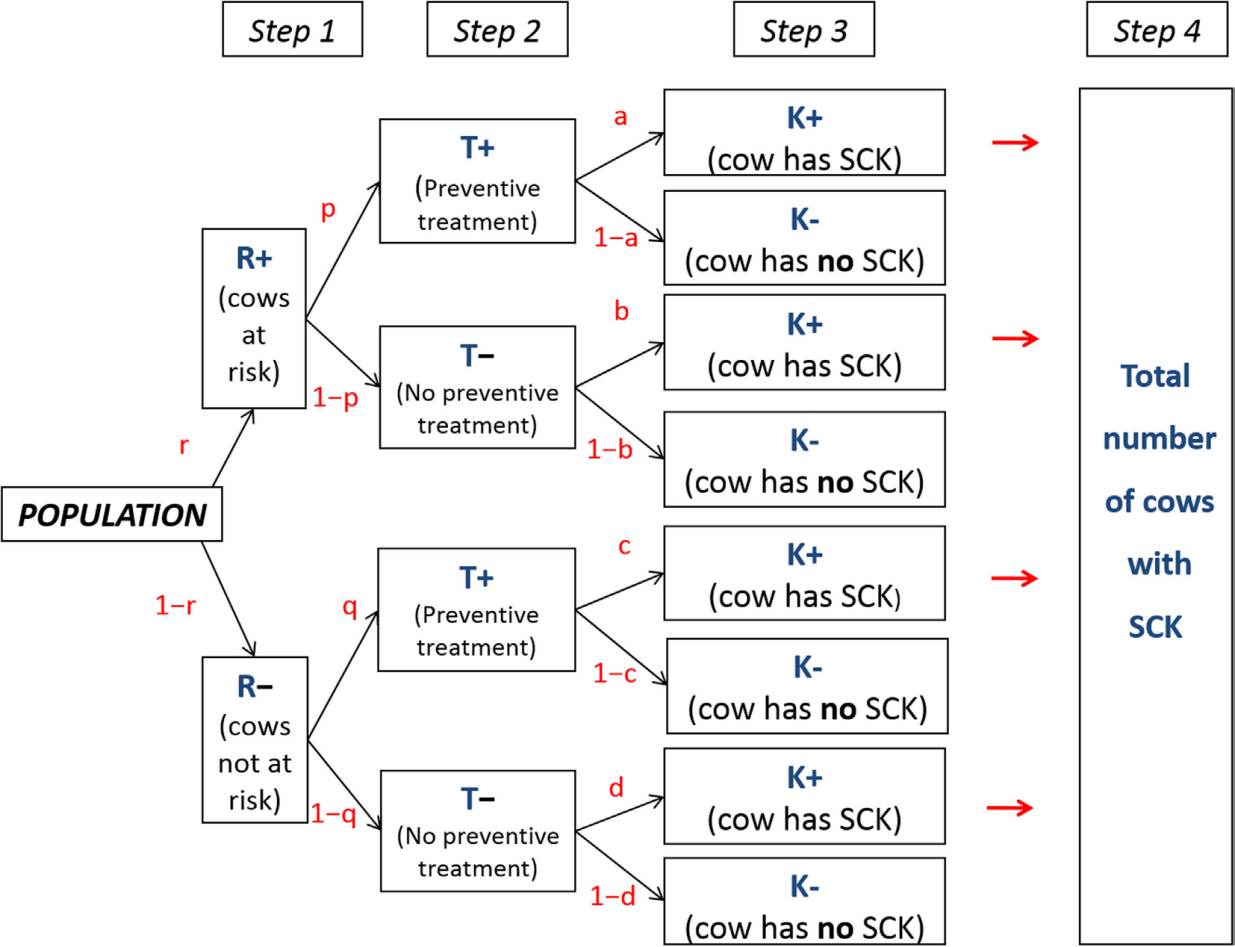
**Conceptual flowchart**. Flowchart representing a conceptual population for which various possible decisions are made. *r* is the prevalence of cows at risk for subclinical ketosis (SCK); *p, q, a, b c*, and *d* are event probabilities; T+ and T− represent a cow that does or does not receive preventative monensin bolus treatment, respectively; and K+ and K− represent a cow with or without SCK, respectively.

A conceptual model aims to simulate a given population and various decisions resulting in different possibilities; such a model is calculated in five successive steps (Figure 1). A similar model was applied previously to assess antimicrobials (6). Briefly, the population included (i) a subpopulation of cows at risk for SCK at a prevalence (*r*), designated the SCK risk prevalence and (ii) a subpopulation of cows not at risk for SCK at a prevalence (1−*r*) (Step 1). Additionally, farmers may utilize a preventative treatment (monensin bolus) on cows at risk for SCK with the probability (*p*) (Step 2). Because farmers do not make perfect decisions, they may also treat a cow not at risk for SCK with a risk (*q*). A scenario lacking any preventative monensin bolus treatment was accounted for within the model with *p* = *q* = 0 (Figure 1). Then, the following denotations were made:

*rp*: the prevalence of cows at risk for SCK and preventatively treated.
*r*(1 − *p*): the prevalence of cows at risk for SCK and not preventatively treated.
(1 − *r*)*q*: the prevalence of cows not at risk for SCK and preventatively treated.
(1 − *r*)(1 − *q*): the prevalence of cows not at risk for SCK and not preventatively treated.

The following step (Step 3) focused on the risk of a cow actually developing SCK (Figure 1). The following denotations were used: *a*, the risk of SCK for a cow at risk of SCK but preventatively treated; *b*, the risk of SCK for a cow at risk of SCK and not preventatively treated; *c*, the risk of SCK for a cow not at risk of SCK but preventatively treated; *d*, the risk of SCK for a cow not at risk of SCK and not preventatively treated.

The parameters *b* and *d* are related by Eq. 1.

(1) $$b=d\times {\text{RR}}_{\text{SCK IF AT RISK}}$$

where RR_SCK IF AT RISK_ is the relative risk of having SCK if risk factors are present in a given cow.

The parameters *a* and *b* are related by Eq. 2.

(2) $$a=b\times \left(\text{1}-{\text{EFF}}_{\text{MONENSIN}}\right)$$

where EFF_MONENSIN_ is the reduction in the prevalence of SCK due to monensin bolus use when administered to cows at risk for SCK.

The parameters *d* and *a* are related by Eq. 3 through Eqs 1 and 2.

(3) $$a=d\times {\text{RR}}_{\text{SCK IF AT RISK}}\times \left(\text{1}-{\text{EFF}}_{\text{MONENSIN}}\right)$$

The parameter *c* was defined as *d*, meaning that the bolus has no efficacy on cows not at risk for SCK. The parameter *d* (risk of SCK for a cow not at risk for SCK and not preventatively treated with monensin) represents the baseline “natural” risk of SCK in cows not exposed to risk factors. The overall prevalence of SCK (*P*_SCK_) and the number of preventative treatments (*N*_TRT_) in the studied population were defined using Eqs 4 and 5 (Step 4).

(4) $$P_{\text{SCK}}=rpa+r\left(\text{1}-p\right)b+\left(\text{1}-r\right)qc+\left(\text{1}-r\right)\left(\text{1}-q\right)d$$

(5) $$N_{\text{TRT}}=rp+\left(\text{1}-r\right)q$$

A targeting index (TI) was then defined according to Eq. 6, which represents the ability of the decision maker to only treat cows at risk.

(6) TI=rp+(1−r)q

The outcome variable of the model was the total cost associated with SCK management (TotalCost_SCK_) at the herd level (Eq. 7).

(7) $${\text{TotalCost}}_{\text{SCK}}={\text{Cost}}_{\text{TRT}}+{\text{Cost}}_{\text{SCK}}=N_{\text{TRT}}\times C_{\text{TRT}}+P_{\text{SCK}}\times C_{\text{SCK}}$$

where Cost_TRT_: the total cost of the monensin boluses for a given herd and situation; Cost_SCK_: the total cost of SCK for a given herd (linked to choices made); *C*_TRT_: the price of one monensin bolus; and *C*_SCK_: the price of one case of SCK.

The model was run using Scilab open source software^2^ with 10,000 iterations, and 95% prediction intervals (PIs) and 95% confidence intervals (CIs) were calculated. PIs facilitate the prediction of the situation for the next farm with 95% probability and CIs indicate the situation in 95 of 100 farms visited.

### Calibration

The key input parameters of the models are summarized in Table 1. The majority of the input parameters were included as a law of distribution (normal or log-normal) and not as a point estimate. The value of the parameter *d* was calculated according to previously defined methods (6): the obtained value of *d* was approximately 15% in the majority of cases and 10% in rare situations (Table 1). RR_SCK IF AT RISK_ was defined according to a meta-analysis (11) conducted on the outcomes “parity” and “body condition score” (BCS). The literature including methods, results, and discussion of the meta-analysis are presented in Text S1 and Table S1 in Supplementary Material. The results of the meta-analysis suggested difficulties in precisely defining RR_SCK IF AT RISK_. Two scenarios were then proposed, in which RR_SCK IF AT RISK_ was defined through LN (0.76, 0.60) or LN (1.50, 0.62) (Table 1). EFF_MONENSIN_ was evaluated according to previously defined methods (6), based on a report on monensin bolus use by the Committee for Medicinal Products for Veterinary Use from the European Medicines Agency^3^ and from four other studies (9, 10, 12, 13). Three values of EFF_MONENSIN_ (Table S2 in Supplementary Material) were retained (Table 1). *C*_SCk_ was previously defined (7). The most plausible, but also the maximal and minimal estimations, were retained from *C*_SCk_. All models were run for an average 100-head dairy cow herd.

**Table 1:** **Input parameters for the model**.

	Law	Main scenario	Sensitivity analysis	Reference
*d*	*N*	0.15 (0.04)^a^	0.10 (0.03)^a^	Raboisson et al. (6)
RR_SCK IF AT RISK_	LN	0.76 (0.60)^b^ or 1.50 (0.62)^b^		Raboisson et al. (6)
EFF_MONENSIN_	//	0.66	0.45 and 0.85	Raboisson et al. (6)
*C*_SCK_, € per cow	LN	257 (94)	177 (82) and 434 (123)	Raboisson et al. (7)
*C*_TRT_, € per bolus	LN	36 (3)		

*LN, log-normal; N, normal*.

*^a^Mean (and SD)*.

*^b^Corresponding to RR_SCK IF AT RISK_ = 2.0 and 4.5*.

*d is the risk of subclinical ketosis (SCK) for a cow not at risk for SCK and not preventatively treated; RR_SCK IF AT RISK_ is the relative risk of having SCK if risk factors are present in a given cow; EFF_MONENSIN_ is the reduction in the prevalence of SCK due to monensin bolus use when administered to cows at risk for SCK; *C*_SCK_ is the price of one case of SCK; and *C*_TRT_ is the price of one monensin bolus*.

## Results

**Figure 2:**
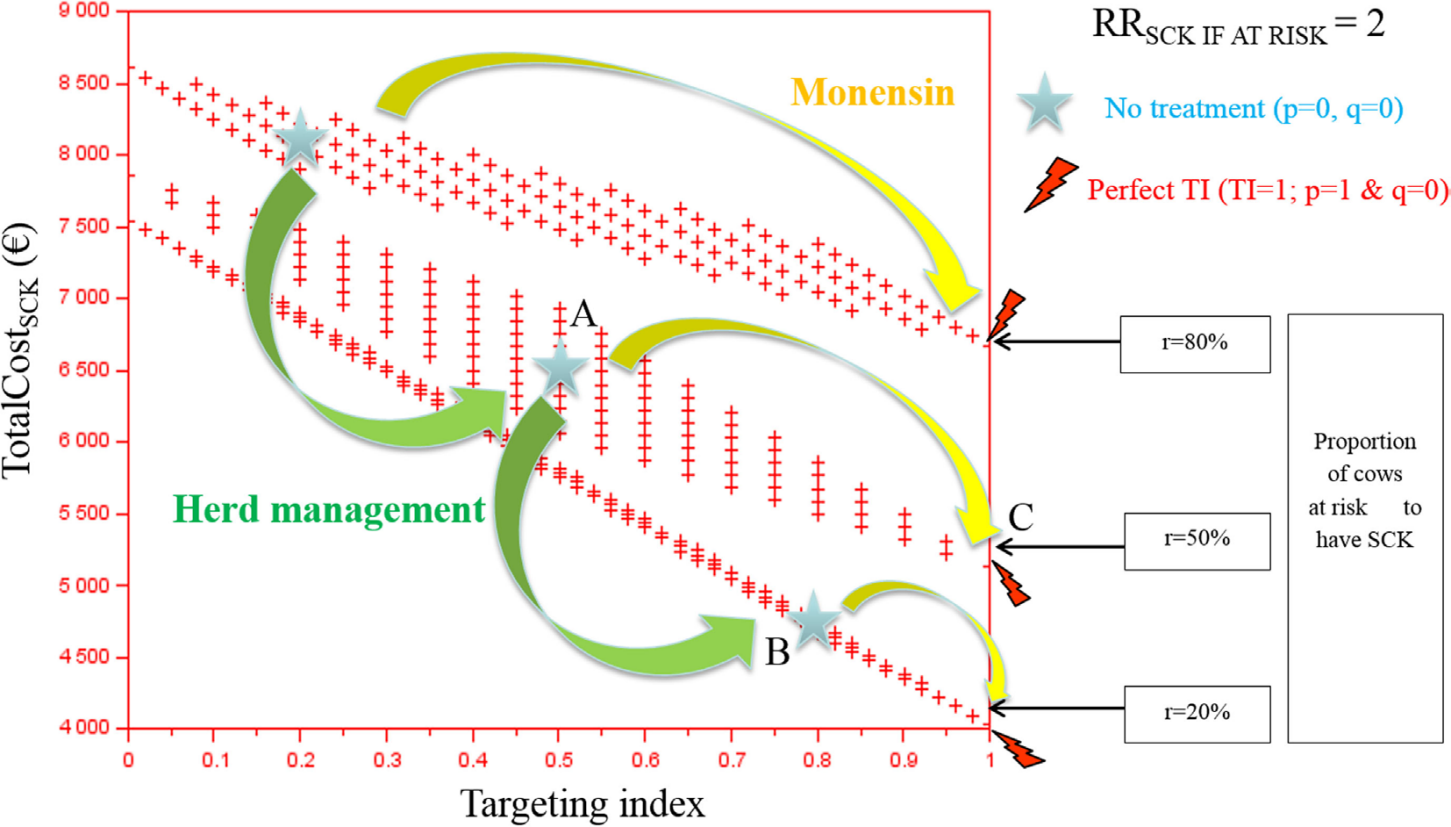
**The total cost of subclinical ketosis (SCK) management (TotalCost_SCK_) for different targeting index (TI) values and three hypothetical prevalences of cows at risk for SCK (*r*) when RR_SCK IF AT RISK_ = 2**. RR_SCK IF AT RISK_ is the relative risk of having SCK if risk factors are present in a given cow; *p* is the proportion of cows at risk for SCK that are preventatively treated with monensin; and *q* is the proportion of cows not at risk for SCK that are preventatively treated with monensin. The graph is read as follows: for a given risk prevalence (for example, *r* = 50%), going from a situation without any preventative treatment (point A) to perfectly targeted preventative treatment (point B) is profitable because it is associated with a decrease in the total cost; without any preventative treatment, the total cost of the disease decreases as the SCK risk prevalence *r* decreases (for example, from *r* = 50 to 20%, meaning from point A to point C).

**Figure 3:**
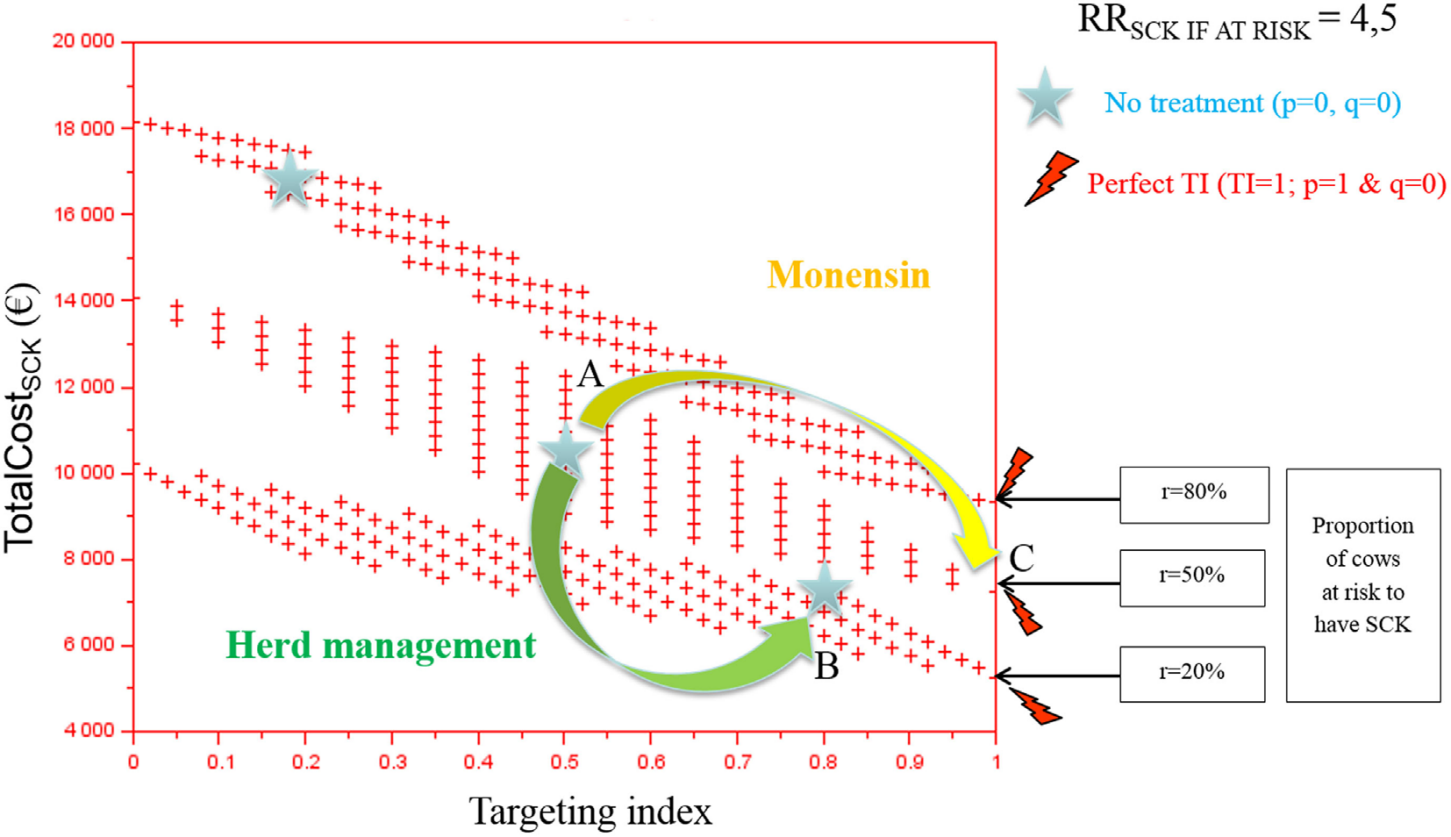
**The total cost of subclinical ketosis (SCK) management (TotalCost_SCK_) for different targeting index (TI) values and three different prevalences of cows at risk for SCK (*r*) when RR_SCK IF AT RISK_ = 4.5**. RR_SCK IF AT RISK_ is the relative risk of having SCK if risk factors are present in a given cow; *p* is the proportion of cows at risk for SCK that have been preventatively treated with monensin; and *q* is the proportion of cows not at risk for SCK that have been preventatively treated with monensin. The graph is read as follows: for a given risk prevalence (for example, *r* = 50%), going from a situation without any preventative treatment (point A) to perfectly targeted preventative treatment (point B) is profitable because it is associated with a decrease in the total cost; without any preventative treatment, the total cost of the disease decreases as the risk prevalence *r* decreases (for example, from *r* = 50 to 20%, meaning from point A to point C).

The TotalCost_SCK_ was first reported for various TI and three values of *r* (Figures 2 and 3). Each cross represents one combination of *p* and *q* values (increment of 0.1 unit). As expected, the TotalCost_SCk_ decreased when *r* decreased in the absence of any preventative treatment (stars in Figures 2 and 3). In other words, reducing the value of *r* by modifying herd management decreased the TotalCost_SCK_ [for example, point A (*r* = 50%) to B (*r* = 20%)], particularly when the value of RR_SCK IF AT RISK_ was high (Figure 3 compared with Figure 2). The change in the TotalCost_SCk_ when *r* decreased represented the expected reduction in losses, i.e., saved losses, but did not include the extra costs associated with changes in herd management to achieve the new (lower) value of *r*. For a given value of *r*, the prevention of SCK with monensin bolus use was profitable (for example, point A to C for *r* = 50%) provided that the TI increased, indicating that the farmer treated only cows at risk for SCK. In cases where it was difficult for the farmer to administer monensin boluses only to cows at risk for SCK (i.e., the TI decreased), monensin bolus use increased the TotalCost_SCk_ compared with no bolus treatment. Therefore, it was not profitable. The change in the TotalCost_SCK_ allowed by monensin bolus administration represented the expected earnings because the TotalCost_SCK_ already included the prevention costs (i.e., the cost of the boluses). Monensin bolus use to prevent SCK decreased the TotalCost_SCK_ to the extent that the TI increased regardless of the value of *r*. For example, up to €1,000 per herd was expected for *r* = 20%, RR_SCK IF AT RISK_ = 4.5, and perfect monensin bolus use (TI = 1) compared with no preventative monensin bolus treatment. The financial situation of the farm was all the more improved by monensin bolus use than the TI was good and the value of *r* was high.

**Figure 4:**
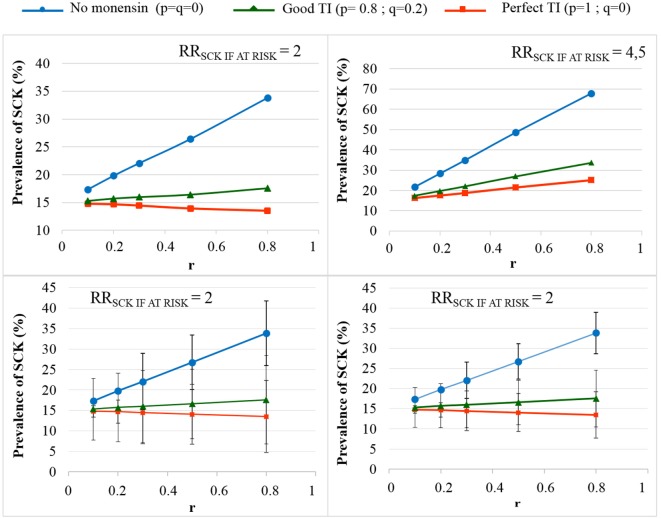
**The prevalence of subclinical ketosis (SCK) for different simulations**. RR_SCK IF AT RISK_ is the relative risk of having SCK if risk factors are present in a given cow; *r* is the prevalence of cows at risk for SCK; targeting index (TI) is the targeting index; *p* is the proportion of cows at risk for SCK that have been preventatively treated with monensin; and *q* is the proportion of cows not at risk for SCK that have been preventatively treated with monensin. The mean values are presented for the main scenario for two values of RR_SCK IF AT RISK_ and three situations involving monensin use: no use, good TI (20% error rate in targeting cows), and perfect TI (no error in targeting cows). The 95% (bottom left) and 80% (bottom right) PIs are represented for RR_SCK IF AT RISK_ = 2.

**Figure 5:**
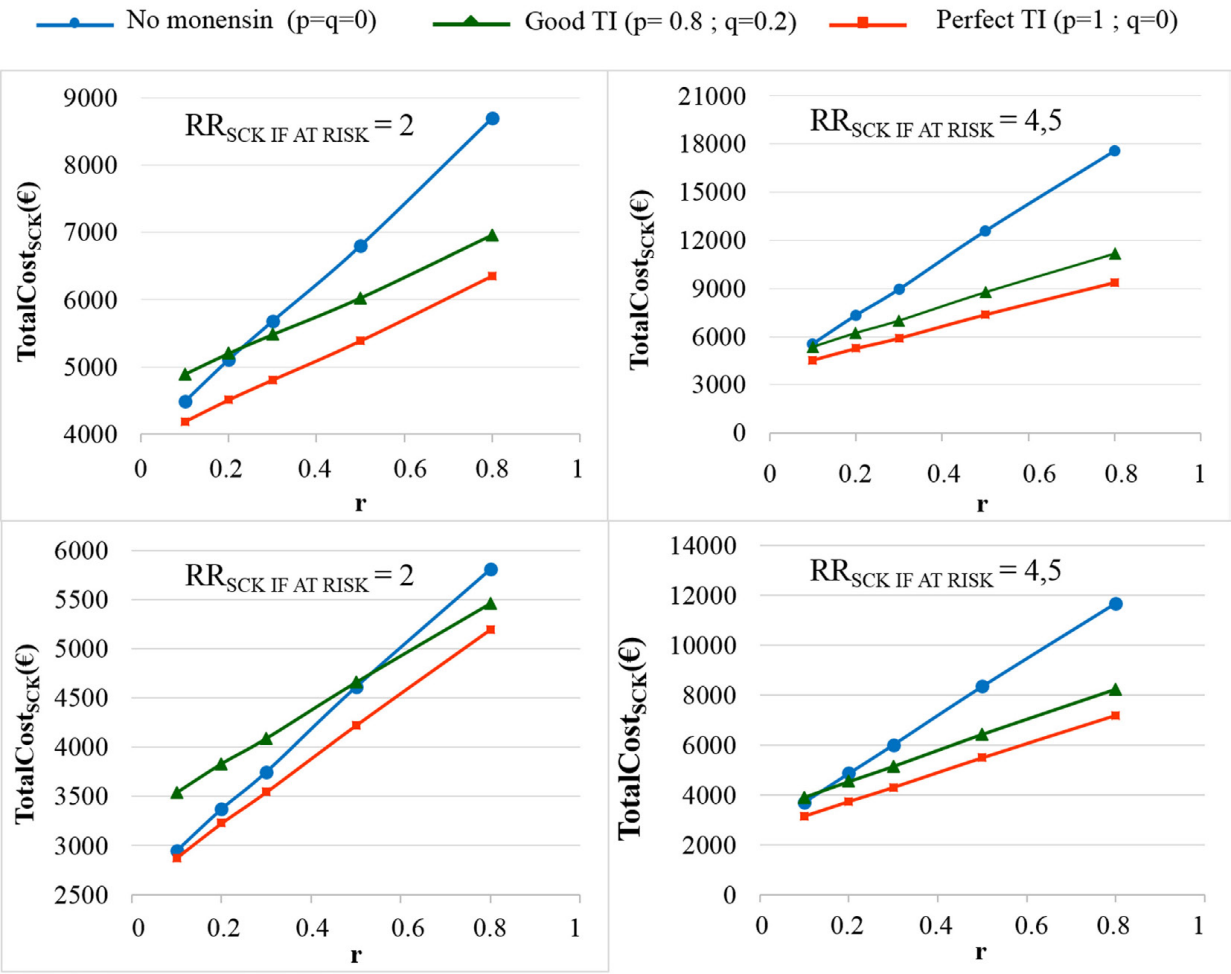
**The total cost of subclinical ketosis (SCK) management (TotalCost_SCK_) for different simulations**. RR_SCK IF AT RISK_ is the relative risk of having SCK if risk factors are present in a given cow; *r* is the prevalence of cows at risk for SCK; TI is the targeting index; *p* is the proportion of cows at risk for SCK that have been preventatively treated with monensin; and *q* is the proportion of cows not at risk for SCK that have been preventatively treated with monensin. The mean values are presented for the main scenario for two values of RR_SCK IF AT RISK_, *d* = *N*(0.15, 0.04) (top) or *d* = *N*(0.10, 0.03) (bottom), and three situations involving monensin use: no use, good TI (20% error rate in targeting cows), and perfect TI (no error in targeting cows).

**Figure 6:**
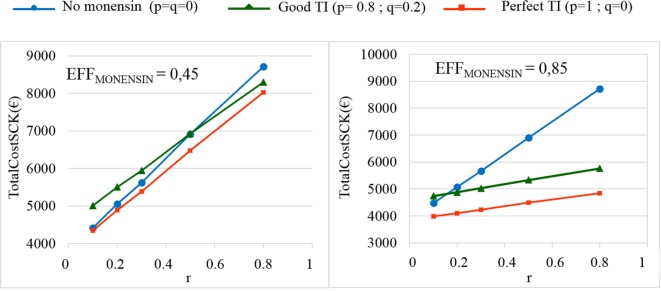
**The total cost of subclinical ketosis (SCK) management (TotalCost_SCK_) for different simulations in which EFF_MONENSIN_ changes**. EFF_MONENSIN_ is the reduction in the prevalence of SCK due to monensin bolus use when administered to cows at risk for SCK; *r* is the prevalence of cows at risk for SCK; TI is the targeting index; *p* is the proportion of cows at risk for SCK that have been preventatively treated with monensin; and *q* is the proportion of cows not at risk for SCK that have been preventatively treated with monensin. The mean values are presented for the main scenario in which RR_SCK IF AT RISK_ = 2 (top) and three situations involving monensin use: no use, good TI (20% error rate in targeting cows), and perfect TI (no error in targeting cows).

**Figure 7:**
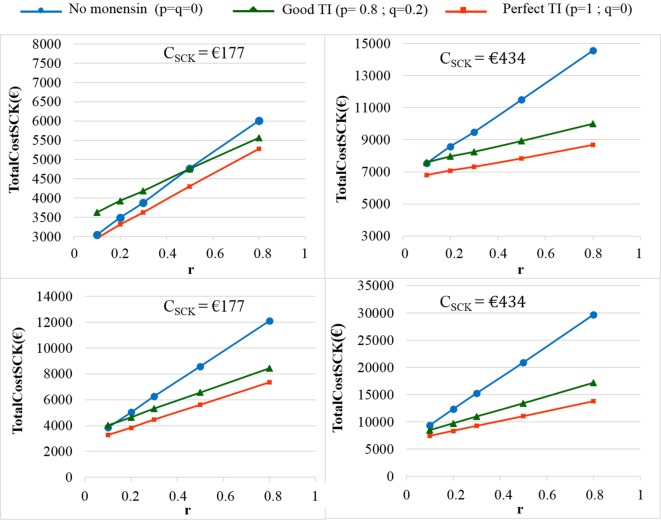
**The total cost of subclinical ketosis (SCK) management (TotalCost_SCK_) for different simulations when the unit total cost of SCK (*C*_SCK_) changes**. *r* is the prevalence of cows at risk for SCK; TI is the targeting index; *p* is the proportion of cows at risk for SCK that have been preventatively treated with monensin; and *q* is the proportion of cows not at risk for SCK that have been preventatively treated with monensin. The mean values are presented for the main scenario in which RR_SCK IF AT RISK_ = 2 (top) or 4.5 (bottom) and three situations involving monensin use: no use, good TI (20% error rate in targeting cows), and perfect TI (no error in targeting cows).

The TotalCost_SCK_ was then reported for different values of *r* and for no monensin bolus use, a perfect TI or a good TI (less than a 20% error rate in targeting). Since targeting cows at risk for SCK is difficult and farmers are unlikely to make perfect decisions when targeting cows, a good TI (20% error rate for *p* and *q*) was considered in addition to a perfect TI. In the main scenario, without any preventative medical treatment (*p* = *q* = 0), the average *P*_SCK_ decreased from 33.8 to 17.2% (OR_SCK IF AT RISK_ = 2) and from 67.9 to 21.7% (OR_SCK IF AT RISK_ = 4.5) when *r* decreased from 0.8 to 0.1 (Figure 4). Perfect monensin bolus use (TI = 1, or *p* = 1, *q* = 0) or correct use (*p* = 0.8 and *q* = 0.2) led to the stabilization of *P*_SCK_ to approximately the financial situation observed for the low value of *r*. Additionally, *P*_SCK_ decreased slightly for a perfect TI and a RR_SCK IF AT RISK_ = 2. The TotalCost_SCK_ was always lower for a perfect TI compared with no preventative treatment or a good TI, as indicated in Figures 5–7. The break-even point was defined as the value of *r* below which a routine (perfect TI or good TI) was associated with a lower TotalCost_SCK_ compared with the lack of preventative treatment; this is the value of *r* where the two lines cross on the graphs. The values of the break-even points were always near zero for the perfect TI (Table 2). This clearly demonstrates the profitability of monensin bolus use, regardless of the SCK risk prevalence and provided that targeting of the cows by the farmer was perfect. The profitability of monensin bolus use remained high in the majority of situations with a good TI. Monensin bolus use was not profitable in cases with a good TI and a low value of *r*, as demonstrated by the high break-even points (Table 2), all the more than EFF_MONENSIN_ was low (Figure 6), *C*_SCK_ was low (Figure 7) and the RR_SCK IF AT RISK_ = 2.

**Table 2:** **Break-even points compared with no preventative treatment with monensin**.

				Break-even point (value of *r*)			
				RR_SCK IF AT RISK_ = 2		RR_SCK IF AT RISK_ = 4.5	
*c*	*d*	EFF_MONENSIN_	*C*_SCK_	Good TI	Perfect TI	Good TI	Perfect TI
=*d*	0.1	0.45	257	0.51	0.02	0.14	0.00
=*d*	0.1	0.66	257	0.24	0.00	0.08	0.00
=*d*	0.1	0.85	257	0.16	0.00	0.06	0.00
=*d*	0.1	0.66	177	0.50	0.02	0.13	0.00
=*d*	0.1	0.66	434	0.11	0.00	0.05	0.00
≠*d*^a^	0.1	0.66	257	0.17	0.00	0.00	0.00
*d*	0.15	0.66	257	0.55	0.02	0.12	0.00

*^a^*c* = *d*(1 − EFF_MONENSIN_/2), i.e., the ability of monensin bolus use to limit the occurrence of SCK fixed at half for cows not at risk for SCK compared with cows at risk for SCK; the break-even points represent the value of *r* below which the strategy involving no preventative treatment was the most profitable; *r* is the prevalence of cows at risk for SCK; RR_SCK IF AT RISK_ is the relative risk of having SCK if risk factors are present in a given cow; EFF_MONENSIN_ is the reduction in the prevalence of SCK due to monensin bolus use when administered to cows at risk for SCK; *C*_SCK_ is the price of one case of SCK; and targeting index (TI) measures whether the farmer treats only the cows at risk for SCK*.

**Figure 8:**
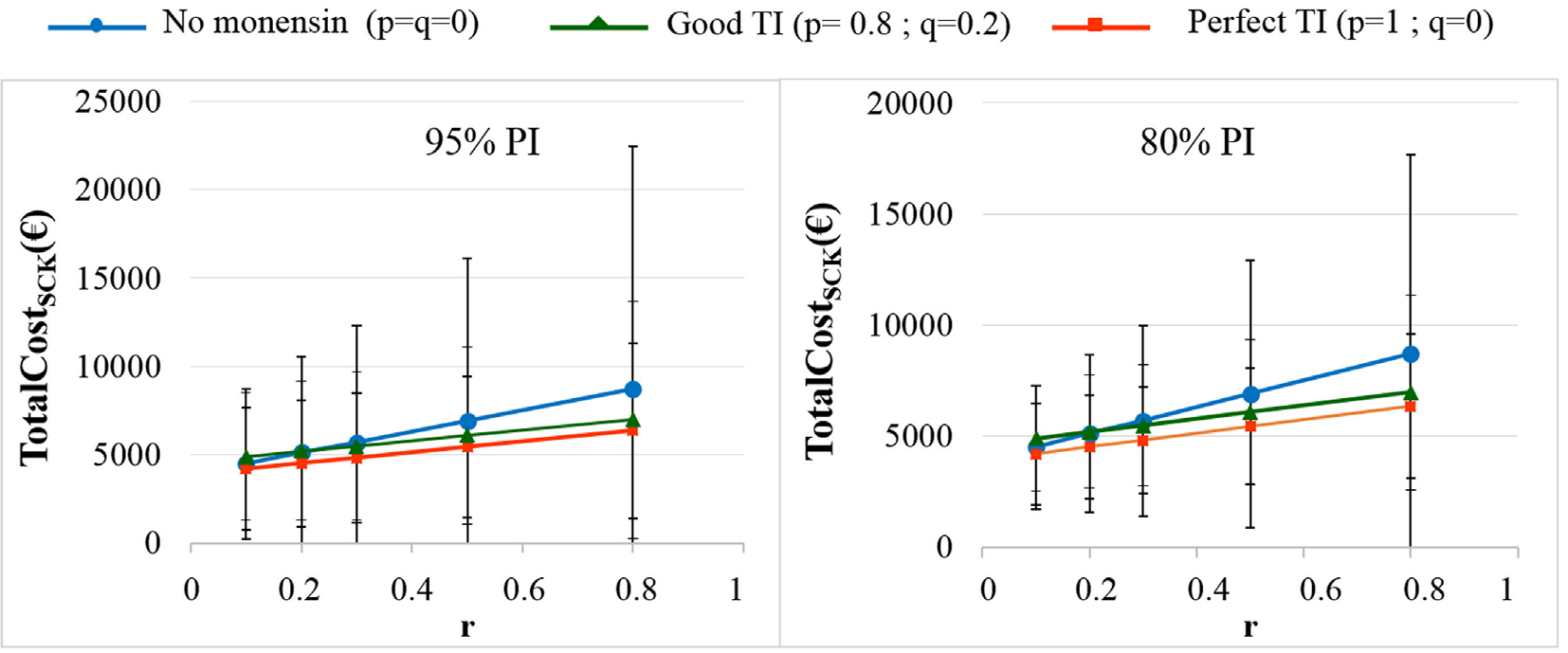
**The total cost of subclinical ketosis (SCK) management (TotalCost_SCK_) for different simulations when RR_SCK IF AT RISK_ = 2**. *r* is the prevalence of cows at risk for SCK; TI is the targeting index; *p* is the proportion of cows at risk for SCK that have been preventatively treated with monensin; and *q* is the proportion of cows not at risk for SCK that have been preventatively treated with monensin. The mean values and their 95% (left) and 80% (right) PIs are presented for the main scenario and three situations involving monensin use: no use, good TI (20% error rate in targeting cows), and perfect TI (no error in targeting cows).

As expected, the herd-level profitability of monensin bolus use was particularly important when the value of *r* was high, as demonstrated by the gap between the lines on the graphs. Optimal economic situations, as defined as the lowest TotalCost_SCK_, were observed for the lowest values of *r*, independent of monensin bolus use. When the efficacy of monensin bolus use on cows not at risk for SCK was limited (i.e., *c* ≠ *d*), the TotalCost_SCK_ did not change substantially (data not shown), and the TotalCost_SCK_ and the break-even points were slightly decreased (Table 2). When the value of *d* was fixed at *N*(0.10, 0.03) (Figure 5, bottom) instead of *N*(0.15, 0.04) (Figure 5, top), the results were unchanged, but the ranges of variation for the TotalCost_SCK_ were reduced, and the values of the break-even points were higher than the values when RR_SCK IF AT RISK_ = 2. The 95% CI ranges for all results were very narrow, indicating the results were precise. The 95 and 80% PI ranges indicated it was difficult to predict the expected changes in the TotalCost_SCK_ for the next population of 100 heads (Figure 8). However, the expected prevalence of SCK was predicted with moderate precision (Figure 4).

## Discussion

### Decreasing the Prevalence of Cows at Risk for SCK and Monensin Bolus Use Are Both Profitable and Synergic Strategies

The present work analyzed the profitability of various scenarios regarding the management of SCK in the field. These options should be seen as a mix of the decisions made by a farmer or farm advisers and of the present situation in a herd. The results clearly showed synergy between a reduction in the prevalence of cows at risk for SCK (value of *r*) and monensin bolus use to reduce the occurrence of SCK in cows already at risk for SCK. Four situations may be considered.

First, if the farmer has a high number of cows at risk for SCK during the mid-dry period, monensin bolus use is profitable and significantly reduces the economic impact due to SCK. This conclusion remained valid irrespective of the calibration retained. The economic relevance of this strategy (monensin bolus use) is in accordance with the constraints observed in the field because there is no other satisfactory solution at this stage to manage the problem in these cows. The profitability of monensin bolus use was demonstrated for high or low values of *r*; however, the stakes are different between these two situations. Monensin bolus use when the prevalence of cows within a herd at risk for SCK is high is only financially justified as a short-term strategy since the economic optimum is not reached. Therefore, reducing the value of the SCK risk prevalence (*r*) is of economic interest.

Second, reducing the value of the SCK risk prevalence (*r*) is associated with a decrease in the TotalCost_SCK_. This was expected since the cost associated with changes in the management of a dairy herd to achieve a low value of *r* was not included in the TotalCost_SCK_. A decrease in the TotalCost_SCK_ due to a decrease in the SCK risk prevalence (*r*) should be compared with the expected costs associated with changes in diet or management before making any decisions. However, these costs are difficult to estimate. Profitability of the decrease in the value of SCK risk prevalence (*r*) is only observed when the cost of diet improvement is low, which is expected since the dietary requirements of dry cows are simple, and imbalance is often a result of excess diet and not diet deficiency. Comparison between the expected saved losses due to the reduction in SCK risk prevalence (*r*) and the expected extra costs necessary to improve the diets of dry cows suggests that this strategy is profitable in the majority of herds. This statement has yet to be verified by the end-users of the present results before any decision to be made in a farm.

Third, based on the break-even points, monensin bolus use was minimally profitable in herds with a low SCK risk prevalence. The break-even points, which indicate the value of SCK risk prevalence below which it is profitable to use monensin boluses, were often low for good TI and were nearly equal to zero for perfect TI. Taken together, these results suggest that monensin bolus use is a good strategy for low SCK risk prevalences, but the success of this strategy depends on the ability of the farmer to only treat the cows at risk for SCK. The expected saved losses will remain limited.

Fourth, further reductions in the SCK risk prevalence (*r*) in herds that already have a low risk prevalence are technically difficult or impossible to achieve. Such reductions may even worsen the situation, such as in cases of inappropriate changes in the diet of dry cows, which would create new risks for SCK in dry cows that were not initially at risk for SCK. The definition of the prevalence of cows at risk for SCK or the cows with SCK corresponding to the economic optimum is out of the scope of the present work.

In summary, the present work showed that (i) monensin bolus use is profitable in the short-term only is high (herd-level acute situation) when the SCK risk prevalence (*r*) is high, (ii) decreasing the SCK risk prevalence *r* (up to a value to be determined by further research) is a baseline strategy when this SCK risk prevalence is high, and (iii) monensin bolus use when the SCK risk prevalence (*r*) is low is a profitable long-term strategy only if the cows are targeted precisely based on their risk for SCK (by the farmer or other personnel). Further work is required to investigate how to better diagnose cows at risk for SCK at 3 weeks prepartum. In western Europe, cows “fat, old, and previously sick” (meaning with too high BCS compared to expected one, in parity 3 or higher, and with ketosis-related illness the previous lactation), are targeted for the use of the bolus. This should stay the present criteria of decision, waiting for future more precise indicators to identify cows at risk. Considering this and the farmer’s expected ability to target SCK-at-risk animals, the good targeting situation (*p* = 0.8, *q* = 0.2) proposed in the present work seems appropriate to represent the average situation in the field.

The present work did not consider the treatment of all cows in a herd since this is constitutes off-label monensin use in Europe. Preventative monensin bolus use in cows not at risk for SCK is a potential situation in the field that is linked to difficulties in correctly identifying cows at risk for SCK. However, this should not be interpreted as a suggestion to undertake extra-label monensin use.

The *C*_SCK_ was estimated on average to be €257 per cow with SCK. Its sensitivity to the margin over feed cost was low. The *C*_SCK_ was €244, €250, €257, €267, €276, and €289 for a margin over feed cost of €80, €100, €120, €150, €180, and €210 per ton of milk, respectively (7). Comparison of this abovementioned variability in *C*_SCK_ for various margins over feed cost with changes in the TotalCost_SCK_ for various *C*_SCK_ (Figure 8) clearly shows that the conclusions of the present work are still valid for low or high market prices of milk. Furthermore, *C*_SCK_ was included in the present model as a law of distribution. Thus, *C*_SCK_ = *N*(257,94) accounted for a significant portion of the variation in the margin over feed cost usually faced in Europe.

### Methods and Model Calibration

The present work stressed a robust calibration, which is consistent with similar studies (6, 7). First, given the 95% PI, it was difficult to predict the expected TotalCost_SCK_. The prediction remained correct for the prevalence of SCK (Figure 4) and was lower than for the TotalCost_SCK_ due to the large 95% PI observed for *C*_SCK_ (7). Despite the large 95% PI for the TotalCost_SCK_, the probability of achieving the average value reported in the graphs was high. Additionally, the *P*_SCK_ values in the present work agree with the values observed in the field (3–5). Thus, the present model was correctly calibrated in accordance with the efforts made to accurately estimate the different input parameters, including a previous meta-analysis (2), a review of the parameters (7), and complementary work performed for this study (6). Moreover, the value of parameter *d* highly influenced the model because the definitions of *a, b*, and *c* are dependent on *d*. However, the meaning and the validity of the results were not modified by changes in the value of *d*. The value of RR_SCK IF AT RISK_ (2.0 or 4.5) remained imprecise. Factors such as feed access around calving or diet energy density after calving may impact the value of RR_SCK IF AT RISK_. Indeed, the results obtained with a RR_SCK IF AT RISK_ = 2.0 or 4.5 may correspond to the farmer’s decision when fat cows are detected late in the dry period; possible decisions range from (i) no change in practice to (ii) improvements in dietary intake and the precise monitoring of cows identified to be at risk for SCK. Last, the present work focuses on a scenario in which *c* = *d*, i.e., no monensin bolus use on cows not at risk for SCK. As a reduction in the baseline risk for SCK cannot be excluded in cases of preventative monensin bolus treatment of cows not at risk for SCK, a related scenario was also proposed, and the results were very similar to the results obtained with *c* = *d*.

## Conclusion

The present work highlights different economic strategies to manage SCK in the dairy industry in Europe, considering, among others, monensin bolus use. First, monensin bolus use when the SCK risk prevalence (*r*) is high is profitable but only in the short-term as a tool to correct an acute herd-level situation. Additionally, decreasing the SCK risk prevalence (*r*) is of financial interest as a baseline strategy when this prevalence is high. Finally, monensin bolus use when the SCK risk prevalence (*r*) is low is also profitable as a long-term strategy but only in cases where cows are precisely targeted according to their risk of SCK (by the farmer or other personnel). Further work is required to analyze the optimal prevalence of cows at risk for SCK in Europe considering the cost of decreasing this prevalence and also to investigate and define clear indicators to classify cows at risk for SCK as early as 3 weeks prepartum.

## Author Contributions

DR and MB made the conception of the work and the interpretation of the results. They both drafted the manuscript and finally approved it. Both agreed to be accountable for all aspects of the work in ensuring that questions related to the accuracy or integrity of any part of the work are appropriately investigated and resolved.

## Conflict of Interest Statement

The authors’ institution received payment from Elanco France for other work. The present work has not been funded by Elanco.

## Footnotes

^1^http://www.med-vet.fr/

^2^http://www.scilab.org.

^3^http://www.ema.europa.eu.

## References

[1] Duffield TF Lissemore KD McBride BW Leslie KE. Impact of hyperketonemia in early lactation dairy cows on health and production. J Dairy Sci (2009) 92(2):571–80. 10.3168/jds.2008-1507 19164667

[2] Raboisson D Mounie M Maigne E. Diseases, reproductive performance, and changes in milk production associated with subclinical ketosis in dairy cows: a meta-analysis and review. J Dairy Sci (2014) 97(12):7547–63. 10.3168/jds.2014-8237 25306269

[3] Philippe P Raboisson D Prévalence de la cétose subclinique dans les troupeaux bovins laitiers de l’Ouest de la France. Rencontres Recherche Ruminants (2012) 137.

[4] Vanholder T Papen J Bemers R Vertenten G Berge AC. Risk factors for subclinical and clinical ketosis and association with production parameters in dairy cows in the Netherlands. J Dairy Sci (2015) 98(2):880–8. 10.3168/jds.2014-8362 25497823

[5] Berge AC Vertenten G. A field study to determine the prevalence, dairy herd management systems, and fresh cow clinical conditions associated with ketosis in western European dairy herds. J Dairy Sci (2014) 97(4):2145–54. 10.3168/jds.2013-7163 24534510

[6] Raboisson D Barbier M Maigne E. How metabolic diseases impact the use of antimicrobials: a formal demonstration in the field of veterinary medicine. PLoS One (2016) 11(10):e0164200. 10.1371/journal.pone.0164200 27716805 PMC5055344

[7] Raboisson D Mounie M Khenifar E Maigne E. The economic impact of subclinical ketosis at the farm level: tackling the challenge of over-estimation due to multiple interactions. Prev Vet Med (2015) 122(4):417–25. 10.1016/j.prevetmed.2015.07.010 26276398

[8] Ospina PA Nydam DV Stokol T Overton TR. Association between the proportion of sampled transition cows with increased nonesterified fatty acids and beta-hydroxybutyrate and disease incidence, pregnancy rate, and milk production at the herd level. J Dairy Sci (2010) 93(8):3595–601. 10.3168/jds.2010-3074 20655428

[9] Duffield TF Sandals D Leslie KE Lissemore K McBride BW Lumsden JH Effect of prepartum administration of monensin in a controlled-release capsule on postpartum energy indicators in lactating dairy cows. J Dairy Sci (1998) 81(9):2354–61. 10.3168/jds.S0022-0302(98)70126-2 9785226

[10] Duffield TF Sandals D Leslie KE Lissemore K McBride BW Lumsden JH Efficacy of monensin for the prevention of subclinical ketosis in lactating dairy cows. J Dairy Sci (1998) 81(11):2866–73. 10.3168/jds.S0022-0302(98)75846-1 9839228

[11] Viechtbauer W Conducting meta-analyses in R with the metafor package. J Stat Softw (2010) 36(3):1–48. 10.18637/jss.v036.i03

[12] Sauer FD Kramer JK Cantwell WJ. Antiketogenic effects of monensin in early lactation. J Dairy Sci (1989) 72(2):436–42. 10.3168/jds.S0022-0302(89)79125-6 2703566

[13] Thomas EE Poe SE McGuffey RK Mowrey DH Alrich RD Effect of feeding monensin to dairy cows on milk production and serum metabolite during early lactation. J Dairy Sci (1993) 76(Suppl 1):280.

