# Supplementary material for: Economic Synergy between Dry Cow Diet Improvement and Monensin Bolus Use to Prevent Subclinical Ketosis: An Experimental Demonstration Based on Available Literature
Source: Front Vet Sci. 2017 Mar 14;4:35. doi: 10.3389/fvets.2017.00035 (PMC5361659; doi:10.3389/fvets.2017.00035)
Supplement: Supplementary file 1 [file Presentation_1.PDF]

Supplemental material

S1 Text. The detailed methods, results and discussion of the meta-regressions performed to define the value of $RR_{SCK\ IF\ AT\ RISK}$.

Methods

The $RR_{SCK\ IF\ AT\ RISK}$ was defined using a meta-analysis (Viechtbauer, 2010). It was conducted on the outcomes "parity" and "Body Condition Score (BCS)" using the Metafor package of R software (version 3.0.2; R Foundation for Statistical Computing, Vienna, Austria). The detailed methods were previously described (Raboisson et al., 2014). Only the two risk factors of SCK parity and BCS were included in the present meta-analysis. Parity represents the number of previous calvings for a given cow and BCS represents the usual criteria to evaluate the fat deposits of cows (Edmonson et al., 1989). BCS ranges from 1 to 5 points, which are given in ¼ point increments, and cows above 3.75-4 points at calving are considered too fat and at risk of SCK (Edmonson et al., 1989; Lomander et al., 2012; McArt et al., 2013; Vanholder et al., 2015).

Results and discussion

The association between SCK and parity was reported in 12 models from 5 publications, some of which were adjusted for BCS (S2 Table). The heterogeneity of the dataset was high (I^2 = 78% [95% CI = 55-94] and Q statistics $\chi^2 = 45$, df = 11, $P < 0.001$). The intercept of the log-effect size in the mixed-effects model with a random effect of publication was 0.79 (SE = 0.12, P < 0.001), which corresponded to an effect size of 2.22 (95% CI = 1.72-2.84). This is the mean RR for parity > 1 compared to parity = 1, as this definition was used in the included studies. Including the moderators, parity reduced the heterogeneity by 53%, and showed a RR of 2.82 [95%CI = 1.87-4.10] for parity = 3 compared to parity = 2.

The association between SCK and parity was reported in 6 models from 3 publications, all adjusted for parity (S2 Table). The heterogeneity of the dataset was high (I^2 = 88% [95% CI=48-98] and Q statistics $\chi^2 = 40$, df = 5, $P < 0.001$). The intercept of the log-effect size in the mixed-effects model with a random effect of publication was 0.28 (SE = 0.18, P < 0.001), which corresponded to an effect size of 1.27 (95% CI = 0.92-1.77). This is the mean RR for BCS > 3 compared to BCS < 3, as this definition was used in the included studies. Including

the moderators, BCS reduced the heterogeneity by 53% and showed an RR of 1.63 [95% CI =1.04-2.56] for NEC > 4 compared to NEC < 3.

The sensitivity analysis showed no outliers for the meta-regressions (data not shown). Based on these results (RR for risk parity and BCS of 2.82 and 1.63, respectively), two final scenarios were retained (Table 1). The first one was defined as the average of the two main risk factors, accounting for the fact that the raw models were at least partially adjusted by the other variable. The lognormal law (mean and SD), LN (0.76,0.60), was then used for $RR_{SCK\ IF\ AT\ RISK}$ in scenario 1 (corresponding to RR = 2). The second scenario considered additivity within these 2 risk factors because of the partial adjustment of the other variable for the raw data and because other rarely reported risk factors were not included here. For instance, the variable twin birth was not statistically significant when added to parity and BCS to explain the risk of SCK (McArt et al., 2013). Moreover, the interaction between parity and BCS was only statistically significant in models using a given definition of SCK and not with other definitions of SCK (McArt et al., 2013). The lognormal law (mean and SD), LN (1.50,0.62), was then used for the $RR_{SCK\ IF\ AT\ RISK}$ in scenario 2 (corresponding to RR = 4.5).

REFERENCES

Edmonson, A.J., Lean, I.J., Weaver, L.D., Farver, T., and Wesbster, G. (1989). A body condition scoring chart for Holstein dairy cows. *J Dairy Sci* 72(1)**,** 68-78.

Lomander, H., Gustafsson, H., Svensson, C., Ingvartsen, K.L., and Frossling, J. (2012). Test accuracy of metabolic indicators in predicting decreased fertility in dairy cows. *J Dairy Sci* 95(12)**,** 7086-7096. doi: 10.3168/jds.2012-5534.

McArt, J.A., Nydam, D.V., and Oetzel, G.R. (2013). Dry period and parturient predictors of early lactation hyperketonemia in dairy cattle. *J Dairy Sci* 96(1)**,** 198-209. doi: S0022-0302(12)00780-1 [pii]

Raboisson, D., Mounie, M., and Maigne, E. (2014). Diseases, reproductive performance, and changes in milk production associated with subclinical ketosis in dairy cows: a meta-analysis and review. *J Dairy Sci* 97(12)**,** 7547-7563. doi: 10.3168/jds.2014-8237.

Vanholder, T., Papen, J., Bemers, R., Vertenten, G., and Berge, A.C. (2015). Risk factors for subclinical and clinical ketosis and association with production parameters in dairy cows in the Netherlands. *J Dairy Sci* 98(2)**,** 880-888. doi: 10.3168/jds.2014-8362.

Viechtbauer, W. (2010). Conducting meta-analyses in R with the metafor package. *J Stat Softw* 36(3)**,** 1-48.
