# Supplementary material for: Economic Synergy between Dry Cow Diet Improvement and Monensin Bolus Use to Prevent Subclinical Ketosis: An Experimental Demonstration Based on Available Literature
Source: Front Vet Sci. 2017 Mar 14;4:35. doi: 10.3389/fvets.2017.00035 (PMC5361659; doi:10.3389/fvets.2017.00035)
Supplement: Supplementary file 2 [file Presentation_2.PDF]

Supplemental material

S2 Table. The raw data used to calculate the relative risk to really contract SCK for cows at risk for SCK ($RR_{SCK\ IF\ AT\ RISK}$)

Outcome variable			Risk		Covariate			
Variable	Definition[1]	Prevalence[2]	RR[3]	Se[4]	Season	Parity[4]	BCS[4]	Reference
BCS[4]	≤ 3 / > 3		0.67	0.15	1	1	na	(Duffield et al., 1998)
BCS	≥ 4 / < 4		1.58	0.17	1	1	na	(Duffield et al., 1998)
BCS	3.25 to 3.75/≤3	61/22	1.5	0.14	1	1	na	(Vanholder et al., 2015)
BCS	≥ 4 / < 3	17/22	2.7	0.2	1	1	na	(Vanholder et al., 2015)
BCS	Moderate / low	44/32	1.1	0.07	0	1	na	(McArt et al., 2013)
BCS	High / low	24/32	1.2	0.08	0	1	na	(McArt et al., 2013)
Parity[4]	P3/P2		1.59	0.13	1	na	1	(Duffield et al., 1998)
Parity	P3/P1		3.13	0.14	1	na	1	(Duffield et al., 1998)
Parity	P2/P1	28/30	1.3	0.2	1	na	0	(Berge and Vertenten, 2014)
Parity	P3/P1	41/30	2.1	0.4	1	na	0	(Berge and Vertenten, 2014)
Parity	P2/P1	27/25	2.1	0.16	1	na	1	(Vanholder et al., 2015)
Parity	≥P3/P1	48/25	2.8	0.15	1	na	1	(Vanholder et al., 2015)
Parity	P2/P1		2.21	0.36	0	na	0	(Lomander et al., 2012)
Parity	≥P3/P1		4.5	0.33	0	na	0	(Lomander et al., 2012)
Parity	P2/P1		3.29	0.37	0	na	0	(Lomander et al., 2012)
Parity	≥P3/P1		5.92	0.46	0	na	0	(Lomander et al., 2012)
Parity	P2/P1	35/34	1.1	0.18	0	na	1	(McArt et al., 2013)
Parity	≥P3/P1	36/34	1.7	0.13	0	na	1	(McArt et al., 2013)

Na : not applicable ; 1: denoted “at risk condition” / “reference condition” ; 2: denoted “number of cows at risk” / “number of cows not at risk” for the outcome variable ; 3: Relative risk and standard error of the linked log normal law; 4:Parity represents the number of calvings for a given cow and BCS represents the usual criteria to evaluate the fat deposit cows (ref). BCS ranges from 1 to 5 points, is given at ¼of points and cows above 3.75- 4 points of BCS at calving at considered as too fat and at risk of SCK

REFERENCES

Berge, A.C., and Vertenten, G. (2014). A field study to determine the prevalence, dairy herd management systems, and fresh cow clinical conditions associated with ketosis in western European dairy herds. *J Dairy Sci* 97(4)**,** 2145-2154. doi: 10.3168/jds.2013-7163.

Duffield, T.F., Sandals, D., Leslie, K.E., Lissemore, K., McBride, B.W., Lumsden, J.H., et al. (1998). Efficacy of monensin for the prevention of subclinical ketosis in lactating dairy cows. *J Dairy Sci* 81(11)**,** 2866-2873. doi: 10.3168/jds.S0022-0302(98)75846-1.

Lomander, H., Gustafsson, H., Svensson, C., Ingvartsen, K.L., and Frossling, J. (2012). Test accuracy of metabolic indicators in predicting decreased fertility in dairy cows. *J Dairy Sci* 95(12)**,** 7086-7096. doi: 10.3168/jds.2012-5534.

McArt, J.A., Nydam, D.V., and Oetzel, G.R. (2013). Dry period and parturient predictors of early lactation hyperketonemia in dairy cattle. *J Dairy Sci* 96(1)**,** 198-209. doi: S0022-0302(12)00780-1 [pii]

Vanholder, T., Papen, J., Bemers, R., Vertenten, G., and Berge, A.C. (2015). Risk factors for subclinical and clinical ketosis and association with production parameters in dairy cows in the Netherlands. *J Dairy Sci* 98(2)**,** 880-888. doi: 10.3168/jds.2014-8362.
