# Supplementary material for: Economic Synergy between Dry Cow Diet Improvement and Monensin Bolus Use to Prevent Subclinical Ketosis: An Experimental Demonstration Based on Available Literature
Source: Front Vet Sci. 2017 Mar 14;4:35. doi: 10.3389/fvets.2017.00035 (PMC5361659; doi:10.3389/fvets.2017.00035)
Supplement: Supplementary file 3 [file Presentation_3.PDF]

Supplemental material

S3 Table. The raw data used to calculate the reduction in subclinical ketosis (SCK) allowed by monensin bolus ($EFF_{MONENSIN}$)

Coefficient of reduction of the SCK prevalence	$EFF_{MONENSIN}$	Bolus of monensin/ Report submitted to authorities for the selling authorisation	Raw data	Reference
0.34	0.66	Yes / Yes	Trial 1, cumulated prevalence of SCK = 11.5 versus 25.6, based on *Least Square Mean*	(CVMP, 2012)
0.27	0.63	Yes / Yes	Trial 2, cumulated prevalence of SCK = 3.3 versus 12.3, based on *Least Square Mean*	(CVMP, 2012)
0.26	0.74	Yes / Yes	Trial 3, prevalence of SCK at day 2 postpartum = 8.2% versus 32.1% based on BHBA threshold to diagnose SCK of 1.0 mmol/L	(CVMP, 2012)
0.15	0.85	Yes / Yes	Trial 4, prevalence of SCK at day 2 postpartum = 3.0% versus 19.6% based on BHBA threshold to diagnose SCK of 1.4 mmol/L	(CVMP, 2012)
0.46	0.54	Yes / No	$EFF_{MONENSIN}$ calculated on the raw mean of SCK prevalence	(Duffield et al., 1998b)
0.80	0.20	No / No	$EFF_{MONENSIN}$ calculated on mean BHBA between exposed and control groups	(Duffield et al., 1998a)
0.55	0.45	Na / Na	$EFF_{MONENSIN}$ calculated on mean BHBA between exposed and control groups	(Sauer et al., 1989)
0.55	0.45	Yes / No	$EFF_{MONENSIN}$ calculated on mean BHBA between exposed and control groups	(Thomas et al., 1993)

Na: not available ; BHB: Beta-hydroxy butyrate

REFERENCES

CVMP (2012). "CVMP assessment report for Kexxtone (EMEA/V/C/002235)", (ed.) C.f.M.P.f.V. Use. www.ema.europa.eu).

Duffield, T.F., Sandals, D., Leslie, K.E., Lissemore, K., McBride, B.W., Lumsden, J.H., et al. (1998a). Effect of prepartum administration of monensin in a controlled-release capsule on postpartum energy indicators in lactating dairy cows. *J Dairy Sci* 81(9)**,** 2354-2361. doi: 10.3168/jds.S0022-0302(98)70126-2.

Duffield, T.F., Sandals, D., Leslie, K.E., Lissemore, K., McBride, B.W., Lumsden, J.H., et al. (1998b). Efficacy of monensin for the prevention of subclinical ketosis in lactating dairy cows. *J Dairy Sci* 81(11)**,** 2866-2873. doi: 10.3168/jds.S0022-0302(98)75846-1.

Sauer, F.D., Kramer, J.K., and Cantwell, W.J. (1989). Antiketogenic effects of monensin in early lactation. *J Dairy Sci* 72(2)**,** 436-442. doi: 10.3168/jds.S0022-0302(89)79125-6.

Thomas, E.E., Poe, S.E., R.K., M., D.H., M., and Alrich, R.D. (1993). Effect of feeding monensin to dairy cows on milk production and serum metabolite during early lactation *Journal of Dairy Science* 76(Suppl. 1)**,** 280.
